# Theoretical Optical Output Power Improvement of InGaN-Based Violet Laser Diode Using AlGaN/GaN Composite Last Quantum Barrier

**DOI:** 10.3390/nano12223990

**Published:** 2022-11-12

**Authors:** Zhenzhuo Zhang, Jing Yang, Degang Zhao, Feng Liang, Ping Chen, Zongshun Liu

**Affiliations:** 1 State Key Laboratory of Integrated Optoelectronics, Institute of Semiconductor, Chinese Academy of Sciences, Beijing 100083, China; 2 College of Materials Science and Opto-Electronic Technology, University of Chinese Academy of Sciences, Beijing 100049, China; 3 Center of Materials Science and Optoelectronics Engineering, University of Chinese Academy of Sciences, Beijing 100049, China

**Keywords:** InGaN-based laser diodes, optical output power, slope efficiency, quantum barrier, electron leakage, hole injection

## Abstract

Electron leakage has an adverse influence on the optical output power for laser diodes (LDs), especially where the conventional last quantum barrier (LQB) in the multiple quantum well (MQW) active region may cause severe leakage problems. In this article, a composite last quantum barrier (CLQB) composed of p-type doped AlGaN (p-AlGaN) and unintentionally doped GaN (u-GaN) layers is designed to replace the conventional one, for overcoming the problem of electron overflow. Theoretical calculations with LASTIP software demonstrate that CLQB with optimized parameters of Al composition, thickness and p-type doping concentration of the p-AlGaN layer in the CLQB can have a 50% improvement in slope efficiency (SE) compared with the conventional structure LD. This will help to realize a higher optical output power in InGaN-based violet LDs.

## 1. Introduction

InGaN-based LDs have attracted great attention as potential light sources in various applications such as automobile headlamps [1], material processing [2], high-density optical data storage [3] and laser-based TVs [4]. In many applications, for example, in automobile headlamps and material processing, high-power violet LDs are required to obtain bright, long-haul illumination or the quick cutting of thick metals [5]. High-powered InGaN-based LDs are not easy to fabricate, and one of the major factors that results in a lower output light power is electron leakage through the MQW active region. There are a few intrinsic causes for it: Firstly, the unbalanced mobilities for electron and hole. The former is known to be one order of magnitude higher than the latter [6]. Secondly, the conduction band offset in the MQW is small [7], especially for MQWs in InGaN-based violet LDs; the energy gap difference between quantum well (QW) and quantum barriers (QB) is smaller compared to InGaN-based blue or green LDs. The smaller gap difference leads to a shallower QW, and further leads to a higher probability for electrons to overflow. Finally, InGaN-based LDs usually have large threshold current density [8,9,10,11], which exacerbates electron leakage [7]. These factors may decrease the slope efficiency (SE) of LDs and thus high optical output power cannot be easily obtained. 

In order to suppress electron overflow and improve the optical output power, many researchers’ work focused on optimizing the electron blocking layer (EBL) [7,12,13,14,15,16,17,18,19], which was first designed to suppress electron leakage by Nakamura [20]. Many new kinds of EBL design have been proposed. For example, Lee et al. [7] introduced an AlGaN/GaN superlattice (SL) EBL and successfully acquired good electron confinement due to the quantum interference. Zhang et al. [13] utilized an AlGaN/GaN SL EBL of gradual Al mole fraction, which improved the hole injection efficiency and lowered the electron leakage. Le et al. [19] employed a polarization-inverted AlInGaN EBL to improve the LD performance. The common point of these designs is that both the electron leakage suppression and hole injection efficiency are taken into account. These designs did work. However, one nature of the electron leakage is the insufficient limiting ability of QBs, and it may be more effective to directly improve QBs’ electron confinement in the MQW region.

In this paper, a p-AlGaN/u-GaN CLQB is proposed and expected to improve the optical output power of InGaN-based violet LDs. Electron overflow rate (EOR), carrier injection efficiency, effective barrier height, optical confinement factor (OCF) and internal loss (IL) in LDs were studied through a LASTIP simulation program. The effect of Al composition, acceptor doping concentration and the thickness of p-AlGaN were analyzed in detail. It was found that an LD with an optimized p-AlGaN/u-GaN CLQB has a 50% improvement in slope efficiency compared with a conventional LD with a single u-GaN layer QB.

## 2. Design Concept and Simulation Parameters

### 2.1. Design Concept

**Figure 1:**
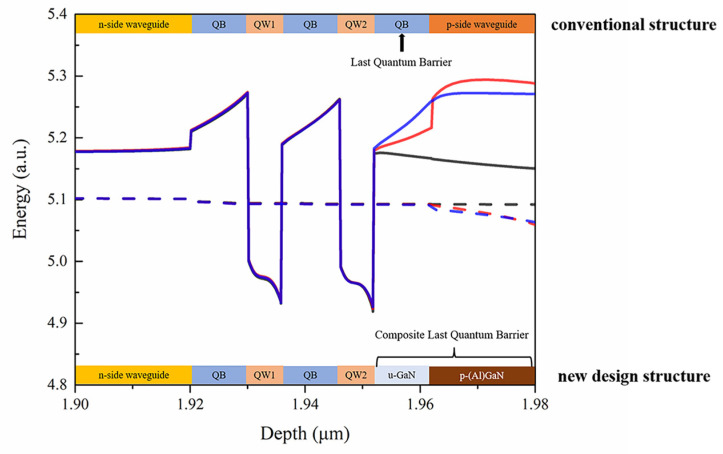
The conduction band diagram of some layers around the MQW region of InGaN-based violet LDs. The conventional structure with u-GaN LQB (black) and the new design structure with p-AlGaN/u-GaN CLQB (red) or p-GaN/u-GaN CLQB (blue). The dashed line represents the electron Fermi level. The energy scale has been vertically shifted to keep the Fermi level consistent.

Figure 1 shows the energy band of several layers around the MQW region at an injecting current of 120 mA in an InGaN-based violet LD. For the LD with a conventional structure, the last quantum barrier (LQB) and the upper waveguide layer (UWG) are composed of a u-GaN layer. As a forward bias is applied on the LD, the electrons will accumulate at the interface of the EBL (which is composed of p-Al_0.2_Ga_0.8_N and is set after the UWG) and u-GaN due to the piezoelectric and spontaneous polarization fields [21]. Thus, the energy band at LQB and UWG are pulled down as shown by the black line in Figure 1, resulting in a condition in which the electron leakage from the second quantum well occurs more easily. In fact, for the InGaN-based violet LD, the layer composition of QWs and QBs is not much different, which exacerbates the leakage.

Our concept is to make the energy band of the LQB bend upwards under forward biasing. The earlier idea was to build a p–n junction, and form a built-in potential which would “elevate” the energy band. However, in many of our simulation attempts, the required doping concentration for p-GaN would be too high (the conduction band diagram of an MQW with p-GaN/u-GaN CLQB is plotted in Figure 1 in the blue line, and the doping concentration of the p-GaN is over 10^20^ cm^−3^); thus, it was not a practical approach for the new LD structure. Therefore, we replaced the p-GaN by p-AlGaN with a low-Al composition (about 5%) to decrease the required doping concentration. Such a designing method finally worked, as shown by the red line in Figure 1. The p-AlGaN/u-GaN CLQB can increase the effective barrier height and enhance the ability of electron blocking, achieving a higher optical output power. In addition to reducing the electron leakage, it was found that the CLQB structure could also assist hole injection, although it also forms a higher barrier in valence band. The explanation will be given at the end of the article.

### 2.2. Simulation Parameters

**Figure 2:**
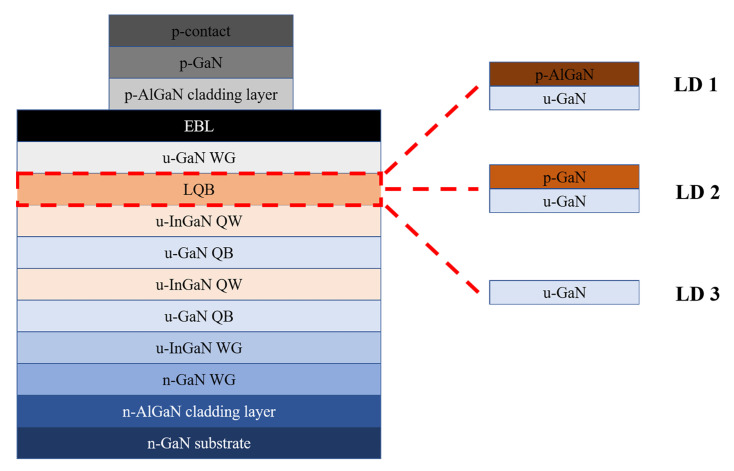
The schematic structure of LDs used in the simulation.

The schematic diagram of the violet MQW LDs for the simulation is shown in Figure 2. The operation characteristics of all LDs were numerically simulated by a LASTIP program (Crosslight Software Inc., Burnaby, BC, Canada), which is a powerful calculation program for photoelectric devices that self-consistently solves the Poisson’s equation and current continuity equations [22,23,24,25,26]. Here, all the layers of the LDs are the same except for the LQB. LD series I represents a series of LDs using a CLQB structure of different Al compositions, thicknesses and doping concentrations of p-AlGaN/u-GaN CLQB. LD1 is a selected one from the LD series Ⅰ. For LD1, the parameters of p-AlGaN in CLQB are 5% (Al composition), 30 nm (thickness) and 2.5 × 10^19^ cm^−3^ (doping concentration). The structure of LD series II is similar to LD series I, except p-AlGaN/u-GaN CLQB is replaced by p-GaN/u-GaN CLQB (the simulation process of LD series Ⅱ is not shown in this article). LD2 is a selected one from the LD series Ⅱ, whose parameters of p-GaN in CLQB are 30 nm (thickness) and 5 × 10^20^ cm^−3^ (doping concentration). LD3 is a conventional violet laser which only uses u-GaN as LQB [22,24,27]. The detailed simulation parameters of each layer of LD1 are listed in Table 1. The band-gap energies of InGaN and AlGaN ternary alloys used for calculation are expressed by the following equations [28]:

(1) $$E_{g}\left(InGaN\right)=0.77x+3.42\left(1-x\right)-1.43x\left(1-x\right)$$

(2) $$E_{g}\left(AlGaN\right)=6.28x+3.42\left(1-x\right)-0.7x\left(1-x\right)$$

where *x* is the composition of Al in AlGaN or In in InGaN. The spontaneous and piezoelectric polarization effects are included in the simulation, and the polarization of ternary nitride alloys can be obtained through a Vegard interpolation of relevant binary compounds with the following expressions [29]:

(3) $$P_{AlGaN}^{sp}=-0.090x-0.034\left(1-x\right)+0.019x\left(1-x\right)$$

(4) $$P_{InGaN}^{sp}=-0.042x-0.034\left(1-x\right)+0.038x\left(1-x\right)$$

(5) $$P_{AlN}^{pz}=-1.808\varepsilon +5.624\varepsilon^{2}\;for\;\varepsilon <0\;$$

(6) $$P_{AlN}^{pz}=-1.808\varepsilon -7.888\varepsilon^{2}\;for\;\varepsilon >0\;$$

(7) $$P_{InN}^{pz}=-1.374\varepsilon +7.559\varepsilon^{2}$$

(8) $$P_{GaN}^{pz}=-0.918\varepsilon +9.541\varepsilon^{2}$$

where *ε* represents the strain. The screening factor of polarization was fixed at 0.25, which means that only 25% of theoretical interface charges are used during the simulation [23]. The operating temperature is assumed to be 300 K. The ionization energy of Si impurity for all materials covered in this article is set as 20 meV, and the ionization energy of Mg impurity for AlGaN is set according to the equation below [30]:

(9) $$I=170\;\left(\mathrm{meV}\right)+3x\;\left(\mathrm{meV}\right)$$

where *x* is the Al composition in AlGaN. The absorption coefficients of n-type and p-type layers were set to 5 and 50 cm^−1^, respectively. When the doping concentration is researched, the free carrier absorption is considered and the absorption coefficients of p-type layers are set according to the following relationship [31]:

(10) $$\alpha_{i}=\frac{doping\;concentration}{10^{19}\;\left(cm^{-3}\right)}\times 25\;\left(cm^{-1}\right)$$

The refractive indexes and activation energy of impurity for AlGaN and InGaN are acquired by the interpolation method according to those of GaN, InN and AlN [32]. The cavity length and ridge width are 600 μm and 3 μm, respectively. The lasing wavelength of all samples is around 405 nm under the fixed 10% In composition of the QW.

## 3. Results and Discussion

### 3.1. Effect of Al Composition in CLQB

**Figure 3:**
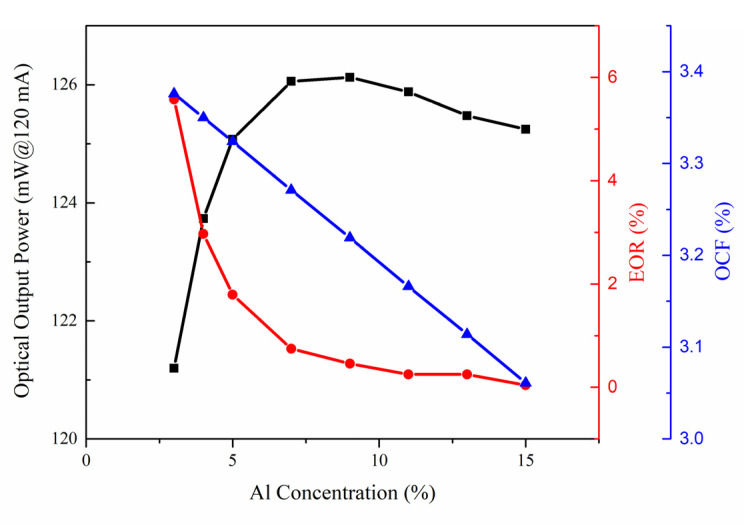
Simulated optical output power (black), electron overflow rate (red) and optical confinement factor (blue) at injecting current of 120 mA versus different Al composition in CLQB of LD series I.

The effect of the Al composition in the AlGaN/GaN CLQB of LD series I is analyzed by varying the Al composition from 3% to 15%. The acceptor doping concentration was fixed at 10^20^ cm^−3^ and the thickness of the AlGaN layer was fixed at 30 nm. The LD output light powers at an injecting current of 120 mA, changing with varying Al composition, are plotted in Figure 3. It is found that the output light power increases rapidly before Al concentration increases and reaches 7%, and afterwards slightly decreases after Al concentration exceeds 9%. The EOR is defined as the ratio of electron current at the position after LQB to one at the position before the first quantum barrier (FQB), and the OCF is the proportion of light in the active region which can be directly acquired from the simulator. It is shown that Al_0.07_Ga_0.93_N/GaN CLQB results in a significantly smaller electron overflow than Al_0.03_Ga_0.97_N/GaN CLQB. As the Al composition continues to increase, the EOR almost approaches 0, indicating that there is almost no leakage current. In the range of Al composition from 3% to 9%, the electron blockage of CLQB improves and thus the optical output power increases. After that, the EOR remains almost unchanged while the OCF keeps decreasing, resulting in a decrease in the output light power. Although a better performance can be obtained when the Al composition reaches 7%, both performance and the difficulty of manufacture should be taken into account. Since InGaN QWs have relatively low thermal stability [33,34], the growth temperature of CLQB is limited. Higher composition AlGaN has poorer quality in actual MOCVD growth at such low temperature [35,36]. Hence, the composition of AlGaN was fixed to be not too high, taking a value at 5% in the following simulation analysis.

### 3.2. Effect of AlGaN Layer Thickness in CLQB

**Figure 4:**
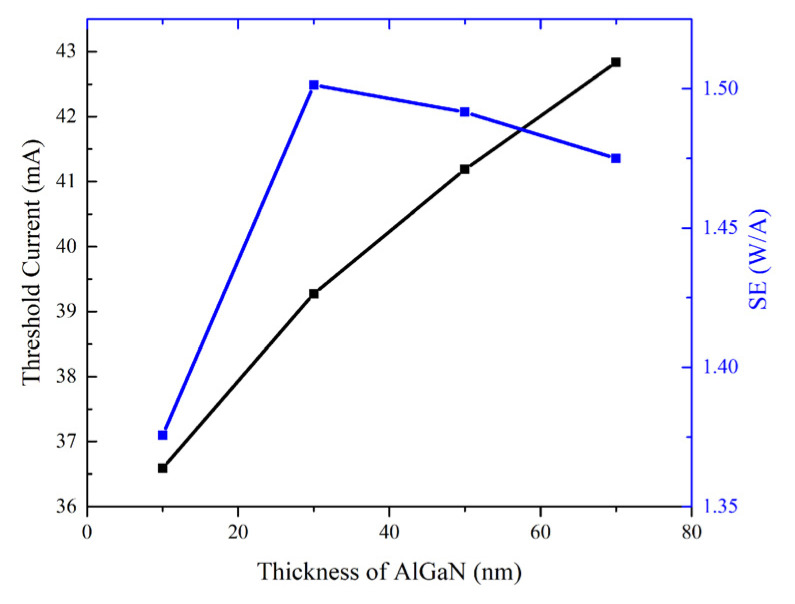
Simulation results for LDs with different thicknesses of the AlGaN layer in CLQB. The threshold current (black) and SE (blue) values are extracted from P–I curves (not shown here). For convenience, the threshold current value is taken when the optical output power reaches 1 mW.

**Figure 5:**
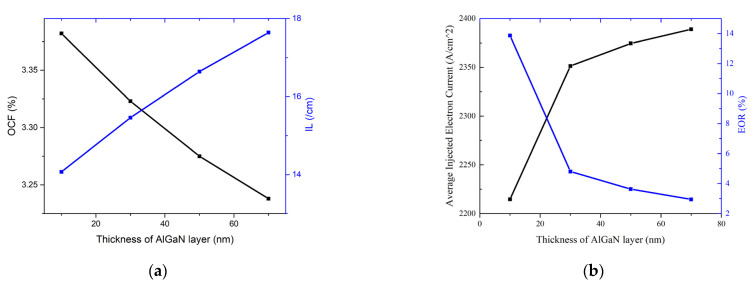
Variations of simulated OCF, IL, average injected electron current and EOR vs. thickness of AlGaN layer in CLQB. (**a**) OCF (black) and IL (blue) vs. thickness of AlGaN layer; (**b**) Average injected electron current (black) and EOR (blue) vs. thickness of AlGaN layer.

The impact of the thickness of the AlGaN layer in CLQB on the LD performance is studied by varying the thickness of AlGaN from 10 nm to 70 nm at a step length of 20 nm. The threshold current and SE can be extracted from the P–I curves, and the results are shown in Figure 4. As shown in Figure 4, with increasing thickness of AlGaN, the threshold current increases and the SE first increases then decreases. The threshold current of LDs largely depends on optical gain and loss [25], and can be obtained by the following equation [5]:

(11) $$J_{th}=\frac{J_{tr}}{\eta_{i}}\cdot \left(\frac{\alpha_{i}+\alpha_{m}}{\Gamma_{v}\cdot G_{0}}\right)$$

where *J_tr_* is the transparent current density, *η_i_* is the internal quantum efficiency (IQE), *α_i_* and *α_m_* are internal loss and mirror loss, *Γ_v_* is the optical confinement factor and *G_0_* is the material gain constant. Gain spectrum, OCF (*Γ_v_*) and IL (*α_i_*) can be directly acquired from the LASTIP calculation, and it is found that the gain near the lasing wavelength (~405 nm) is almost unchanged. *J_tr_* can be extracted from the gain spectrum at a position where the gain is exactly 0, and is basically the same. *η_i_* mainly depends on Shockley–Read–Hall (SRH) non-radiative recombination, spontaneous radiative recombination and Auger non-radiative recombination. Variations in these factors are not considered in the calculation (i.e., the coefficients are set to be consistent), so *η_i_* can also be considered consistent. The relations of OCF and IL vs. the thickness of the AlGaN layer in CLQB are plotted in Figure 5. Due to the high activation energy of the Mg acceptor, p-(Al)GaN always has a low ionization rate, which results in a higher absorption loss in p-type material. Meanwhile, the refractive index of AlGaN is lower compared to GaN and InGaN, and so the average refractive index of the MQW region decreases when the thickness of AlGaN increases. These induce a drop in OCF. The decrease in the OCF and the increase in IL may lead to a higher threshold current.

SE is described by the equation below [37]:

(12) $$SE=\eta_{inj}\cdot \frac{hc}{e\lambda }\cdot \frac{\alpha_{m}}{\alpha_{i}+\alpha_{m}}$$

where *η_inj_* is the carrier injection efficiency, h and c are the Planck constant and speed of light and λ represents the wavelength of the laser. *η_inj_* is greatly affected by EOR and is proportional to the number of injected carriers. The vertical carrier current can be calculated by LASTIP, and the injected current can be obtained by the change in carrier current between two neighboring QBs. Here, we use the injected electron current to reflect the carrier injection efficiency. The relations between average electron injection efficiency and EOR vs. the thickness of the AlGaN layer are displayed in Figure 5. As the thickness of the AlGaN layer increases, the width of the barrier increases, which enhances the ability to block electron overflow and improves the carrier injection efficiency. However, when the thickness increases to reach 30 nm, the role of enhancement is no longer significant. However, IL continues to increase, which may lead to a drop in the SE.

### 3.3. Effect of Acceptor Doping Level in CLQB

**Figure 6:**
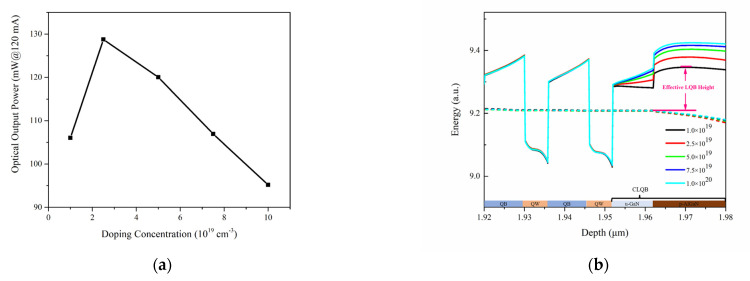
Variation of simulated optical output power and conduction band diagram when different acceptor doping concentration is used for AlGaN layer in CLQB. (**a**) Optical output power at injecting current of 120 mA; (**b**) Conduction band diagram around the MQW region at injecting current of 120 mA. The dashed line represents the electron Fermi level. The doping concentration of p-AlGaN layer in 4 samples is 1.0 (black), 2.5 (red), 5.0 (green), 7.5 (violet) and 10.0 (cyan) × 10^19^ cm^−3^. The energy scale has been vertically shifted to keep the Fermi level consistent, making it easier to tell the difference.

**Figure 7:**
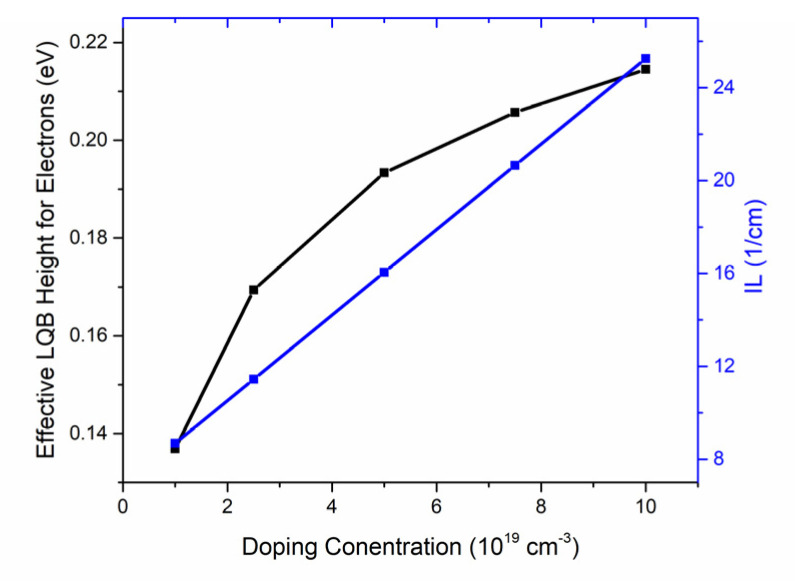
Effective LQB height for electrons (black) and IL (blue) for various acceptor doping concentrations.

The influence of the acceptor doping concentration of the AlGaN layer in CLQB in the range from 1.0 × 10^19^ to 1.0 × 10^20^ cm^−3^ on the LDs is analyzed. The CLQB of these samples has a fixed Al_0.05_Ga_0.95_N layer thickness of 30 nm. As shown in Figure 6a, the optical output power at an injecting current of 120 mA first increases with increasing doping concentration rapidly, then starts to drop. The conduction band diagrams at different doping levels were displayed in Figure 6b. To better compare their differences, the energy bands were vertically shifted to keep the Fermi level the same in the MQWs under different doping concentration conditions. It can be seen that when the acceptor doping concentration increases, the conduction band of the u-GaN part in the CLQB turns from bending downwards to bending upwards and the degree of upward bending increases with the increase in doping concentration. However, the bending of the p-AlGaN part stays almost unchanged. This phenomenon can be explained by p–n junction theory. The built-in potential for a p–n junction can be written as [38]:

(13) $$\phi_{bi}=\frac{kT}{e}\ln\frac{N_{A}N_{D}}{n_{i}^{2}}$$

where *Φ_bi_* is built-in potential, *k* and *e* are the Boltzmann constant and element charge, *T* is temperature, *N_A_* and *N_D_* are doping concentration of the acceptor and donor and *n_i_* is the intrinsic carrier concentration. The barrier height for the p–n junction increases with doping concentration, and the depletion region mainly falls in the side with lower doping level. Hence, the depletion region mainly located at the u-GaN area and its energy band deforms to generate a higher barrier. When the doping concentration is 1.0 × 10^19^ cm^−3^, the built-in field is not enough to counter the polarization field induced by the accumulative electron at the interface of UWG and EBL. Hence, the band still bends downwards, leading to a lower optical output power. With the increase in doping concentration, the built-in field becomes stronger and the barrier for electrons becomes higher. Here, we defined the effective LQB height for electrons as the gap between the highest conduction band level in CLQB and the Fermi level in the last quantum well (e.g., see the arrows marked in Figure 6b). The relationship between LQB height, IL and doping level is shown in Figure 7. The effective LQB height for electrons becomes higher with a higher doping level, indicating a better control of electron overflow. However, with a higher doping level, the IL also increases due to the absorption of unionized Mg impurity, which limits the further improvement of output light power. The variation in SE is the same as the optical output power at an injecting current of 120 mA, and the threshold current increases due to the increasing of internal loss. The analysis of SE and threshold are similar to the analysis in Section 3.2 and therefore are not discussed here.

### 3.4. Comparison between LDs with Different LQB Structures

**Figure 8:**
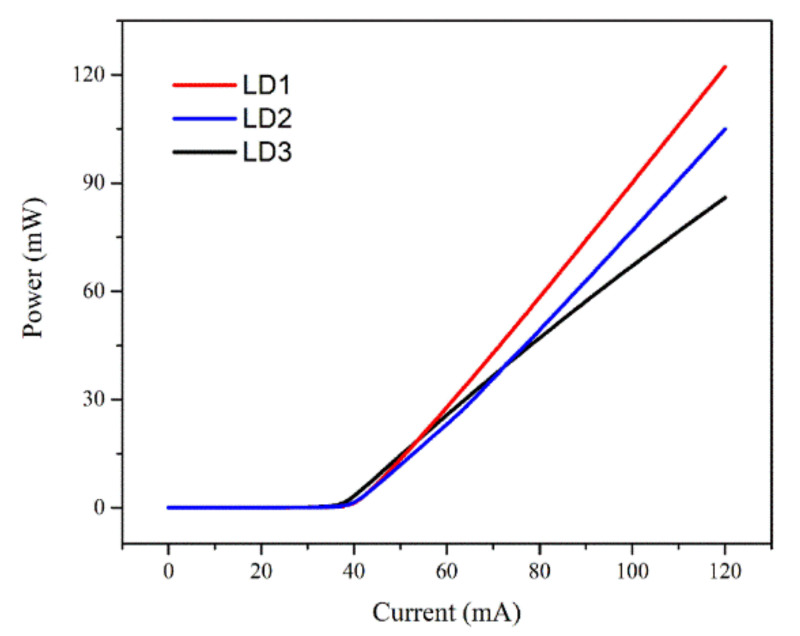
Comparison of simulated P–I curves for three LDs: LD1 with p-AlGaN/u-GaN CLQB (red); LD2 with p-GaN/u-GaN CLQB (blue); LD3 with u-GaN LQB (black).

Jointly considering the obtained simulation results and the practical feasibility of device fabrication, the structure parameters of the final CLQB of AlGaN are chosen as 30 nm-thick, the doping concentration is 2.5 × 10^19^ cm^−3^ and the Al composition is 5%. The LD with a selected p-AlGaN/u-GaN CLQB from LD series Ⅰ is named as LD1. The performances of LDs using p-GaN and u-GaN layers as CLQB have also been studied in a similar way (not shown here), and the selected structure parameters of the p-GaN in CLQB is 30 nm thick and the doping concentration is 5 × 10^20^ cm^−3^. It is noted that its p-type layer is GaN instead of AlGaN, and its p-type doping concentration is one order of magnitude higher than the p-AlGaN layer in p-AlGaN/u-GaN CLQB. The LD with the selected p-GaN/u-GaN CLQB from LD series Ⅱ is named as LD2. In addition, another LD with conventional u-GaN LQB is also studied which is named as LD3. The P–I curves of three LDs, i.e., LD1, LD2 and LD3, are calculated and plotted in Figure 8. The optical output power of LD1 is greatly higher than LD3 by 50% at the injecting current of 120 mA.

**Figure 9:**
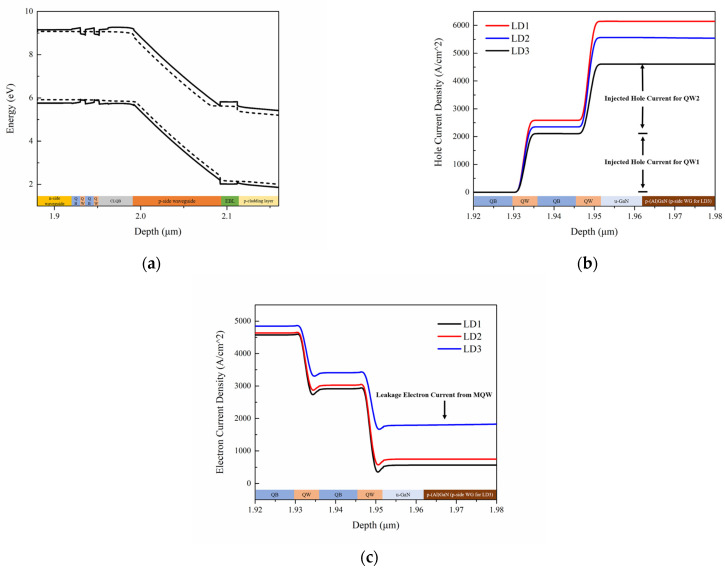
Band energy diagram of LD1 and hole/electron current density around MQW of three LDs. (**a**) Band energy diagram of LD1 at an injecting current of 120 mA where the dashed lines represent electron and hole Fermi levels; (**b**) Hole current distribution around the MQW regions at the vertical direction of three LDs; (**c**) Electron current distribution around the MQW regions at the vertical direction of three LDs.

**Figure 10:**
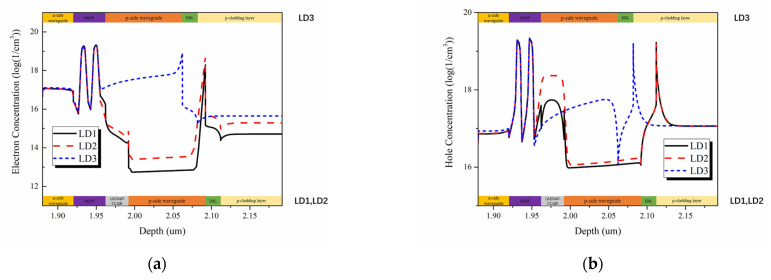
The simulated electron and hole concentration distribution around MQW of three LDs. (**a**) electron concentration distribution at an injecting current of 120 mA; (**b**) hole concentration distribution at an injecting current of 120 mA.

The much better output efficiency of LD1 is due to the fact that besides blocking electron current leakage, the CLQB can also help hole transport. The energy band structure of the LD1 with CLQB at an injecting current of 120 mA is shown in Figure 9a. The hole and electron current density in the MQW region of three LDs are plotted in Figure 9b,c. Obviously, LD1 has the highest hole injection efficiency and the lowest leakage current among the three LDs. Figure 10 shows the carrier concentration distribution near the active region at an injecting current of 120 mA. It is found that the carrier concentration in the MQW keeps unvaried; however, the carrier concentration outside the MQW varies significantly. For electrons, a peak at the interface of UWG and EBL is attributed to the polarization effect. The electron concentration in the UWG decreases due to the introduction of the CLQB structure, indicating that the electron leakage is suppressed. For holes, the accumulation of hole at the interface of EBL and p-cladding layer (and the interface of u-GaN and p-AlGaN in CLQB in LD1) is also attributed to the polarization effect. The hole concentration in the LD with CLQB structure is significantly higher near the MQW. The possible reasons for this phenomenon are as follows: the CLQB raises the band in this region so that the hole transport from p-side to CLQB is easier; furthermore, part of the CLQB is p-type doped. These two factors make the hole concentration higher in CLQB and increase the probability of hole injection from CLQB to MQW. Thus, the hole injection becomes more efficient, although the introduction of AlGaN increases the electron barrier as well as the hole barrier. More data comparisons among the three LDs are listed in Table 2. It is because of the improved carrier injection efficiency (both electrons and holes) that the optical output power of the LD1 with AlGaN/GaN CLQB is greatly increased compared to the conventional InGaN-based violet laser LD3.

## 4. Conclusions

In summary, a series of InGaN-based violet LDs with different LQBs were studied by using the two-dimensional simulator LASTIP. A new LD structure was designed, in which a CLQB composed of p-AlGaN and u-GaN layers was introduced. The influence of Al composition, thickness and the acceptor doping level of the p-AlGaN layer in CLQB on the LD performances were investigated, respectively. The LD with optimized AlGaN/GaN CLQB had a 50% improvement in the SE of the P–I curve compared with the conventional structure LD, which is attributed to a significant reduction in electron leakage and an improvement in hole injection efficiency.

## Figures and Tables

**Table 1:** Simulation parameters of each layer of LD1.

Layers in LD Structure	Al or In Composition (%)	Layer Thickness (nm)	Doping Type	Doping Concentration (cm^−3^)	Dopant
p-contact	0	20	p	1 × 10^20^	Mg
p-GaN	0	50	p	1 × 10^19^	Mg
p-AlGaN cladding layer	7	600	p	5 × 10^18^	Mg
p-AlGaN EBL	20	20	p	1 × 10^19^	Mg
p-side GaN WG	0	100	n	5 × 10^16^	u *
AlGaN/GaN CLQB	5	30	p	2.5 × 10^19^	Mg
	/0	/10	/n	/5 × 10^16^	/u
InGaN QW	10	6	n	5 × 10^16^	u
GaN QB	0	10	n	5 × 10^16^	u
InGaN QW	10	6	n	5 × 10^16^	u
GaN QB	0	10	n	5 × 10^16^	u
n-side InGaN/GaN WG	1	40	n	5 × 10^16^	u
	/0	/80	/n	/2 × 10^17^	/Si
n-AlGaN cladding layer	7	800	n	5 × 10^18^	Si
n-GaN substrate	0	1000	n	1.5 × 10^18^	u

* u represents unintentionally doped with residual donors.

**Table 2:** Comparisons of simulation results among three LDs. For convenience, the threshold current is taken when the optical output power reaches 1 mW.

		LD1	LD2	LD3
CLQB Structure Parameter	Structure	p-Al_0.05_Ga_0.95_N /u-GaN	p-GaN/ u-GaN	u-GaN
	p-type Doping Concentration (10^19^ cm^−3^)	2.5	50	-
	Thickness of p-type Layer (nm)	30	30	-
Performance	Power@120 mA (mW)	128.8	105.0	85.9
	Threshold Current (mA)	36.6	39.0	36.9
	SE (W/A)	1.53	1.28	1.02
	LQB Barrier Height for Electron(meV)	169.4	182.2	79.9
	EOR (%)	7.9	15.7	36.6
	Average Injected Hole Current (A/cm^2^)	2949	2785	2306

## Data Availability

The data that support the findings of this study are available from the corresponding author upon reasonable request.
